# Preventative and therapeutic potential of tocotrienols on musculoskeletal diseases in ageing

**DOI:** 10.3389/fphar.2023.1290721

**Published:** 2023-12-11

**Authors:** Siti Liyana Saud Gany, Kok-Yong Chin, Jen Kit Tan, Amilia Aminuddin, Suzana Makpol

**Affiliations:** ^1^ Department of Biochemistry, Faculty of Medicine, Universiti Kebangsaan Malaysia, Kuala Lumpur, Malaysia; ^2^ Department of Pharmacology, Faculty of Medicine, Universiti Kebangsaan Malaysia, Kuala Lumpur, Malaysia; ^3^ Department of Physiology, Faculty of Medicine, Universiti Kebangsaan Malaysia, Kuala Lumpur, Malaysia

**Keywords:** musculoskeletal, ageing, tocotrienol, muscle ageing, sarcopenia, osteoporosis, arthritis, TRF

## Abstract

Musculoskeletal health is paramount in an ageing population susceptible to conditions such as osteoporosis, arthritis and fractures. Age-related changes in bone, muscle, and joint function result in declining musculoskeletal health, reduced mobility, increased risk of falls, and persistent discomfort. Preserving musculoskeletal wellbeing is essential for maintaining independence and enhancing the overall quality of life for the elderly. The global burden of musculoskeletal disorders is significant, impacting 1.71 billion individuals worldwide, with age-related muscle atrophy being a well-established phenomenon. Tocotrienols, a unique type of vitamin E found in various sources, demonstrate exceptional antioxidant capabilities compared to tocopherols. This characteristic positions them as promising candidates for addressing musculoskeletal challenges, particularly in mitigating inflammation and oxidative stress underlying musculoskeletal disorders. This review paper comprehensively examines existing research into the preventive and therapeutic potential of tocotrienols in addressing age-related musculoskeletal issues. It sheds light on the promising role of tocotrienols in enhancing musculoskeletal health and overall wellbeing, emphasizing their significance within the broader context of age-related health concerns.

## 1 Introduction

Maintaining musculoskeletal health is vital for overall wellbeing and individual vitality. As people age, the risk of musculoskeletal issues like osteoporosis, arthritis, and fractures increases. Preserving musculoskeletal health becomes crucial for sustaining mobility and a good quality of life, especially in the ageing population (Lewis et al., 2019). The natural progression of ageing gives rise to intricate transformations within our bones, muscles, and joints, precipitating a gradual deterioration in musculoskeletal wellbeing. This decline, in turn, manifests as compromised mobility, heightened susceptibility to falls, and persistent, distressing discomfort (Roberts et al., 2016). Furthermore, musculoskeletal health’s significance extends to preserving autonomy in pivotal activities of daily living, encompassing tasks like dressing, bathing and walking. Henceforth, it is imperative to underscore the significance of preserving musculoskeletal health as a pivotal factor influencing the independence and overall quality of life among the elderly. Moreover, the escalating prevalence of age-associated physical incapacitations heralds an imminent surge in healthcare system requisites, underscoring the pressing need for proactive measures to counteract these challenges (World Health Organization, 2017). Musculoskeletal disorders are a formidable global burden, exacting profound tolls on individuals, healthcare infrastructures, and social support systems (Cross et al., 2014).

Based on a recent analysis of the Global Burden of Disease (GBD) 2019 data, a staggering 1.71 billion individuals globally contend with the repercussions of musculoskeletal disorders. (Cieza et al., 2020). The inevitability of skeletal muscle atrophy as a result of the ageing process is indisputable. In this regard, both men and women encounter an annual decline in muscle mass, with men and women, on average, experiencing reductions of 0.47% and 0.37%, respectively, throughout their lifespans, as observed in various studies. (Mitchell et al., 2012). Concurrent with the notable loss of skeletal muscle mass, a substantial decline in strength ensues, exhibiting variability ranging from 0.3% to 4.2% daily (Verdijk et al., 2014). Prominent among the musculoskeletal disorders are osteoarthritis (OA), rheumatoid arthritis (RA), psoriatic arthritis (PsA), gout, lower back pain (LBP), osteoporosis (OP) and sarcopenia (Lewis et al., 2019).

Tocotrienols, lipid-soluble molecules encompassed within the vitamin E family, play a pivotal role in an array of intricate biological processes. These compounds are present in varying quantities within sources such as annatto, palm oil, rice bran, coconut oil, and barley. The vitamin E content of annatto bean is composed of tocotrienol exclusively, while palm oil contains approximately 70% tocotrienols and 30% alpha-tocopherol. Distinguished by unsaturated phytyl side chain isoprenoids, tocotrienols’ distinct structural attributes facilitate their enhanced permeation through saturated fatty tissue layers, thereby underlining their unique transversal capability (Chakraborty et al., 2014). Tocotrienols possess three double bonds within their primary molecular structure, situated at the 3′, 7′ and 11’ positions along the hydrocarbon tail. This configuration imparts enhanced fluidity to tocotrienols and fosters facile absorption into cellular membranes, akin to edible oils characterised by elevated polyunsaturated fatty acid content (Srikaeo, 2014).

Tocotrienols are regarded to be more powerful antioxidants than α-tocopherol (Aggarwal et al., 2010). As evidenced by a spectrum of *in vivo* and *in vitro* investigations, tocotrienol surpasses tocopherol in terms of antioxidant activity owing to factors such as: 1) tocotrienol’s distribution within the lipid membrane is notably more uniform; 2) the presence of double bonds within tocotrienol’s isoprenoid side chain augments its interaction with free radicals; and 3) its heightened redox cycling efficiency serves to enhance its antioxidant prowess further (Packer et al., 2001). Indeed, it has been demonstrated that tocotrienols exhibit superior efficacy compared to tocopherols in thwarting bone loss in animal models (Norazlina et al., 2010). Further investigations have illuminated that the tocotrienol-rich fraction (TRF) outperforms α-tocopherol in its capacity to enhance replicative senescence-associated anomalies and foster myogenic differentiation (Khor et al., 2016). These inherent attributes suggest that tocotrienols hold the potential for mitigating the inflammation and oxidative stress that underlie musculoskeletal disorders.

Research on tocotrienols and their potential impact on musculoskeletal health is still a growing area of interest. A recent review article by Chin et al. touched on the role of vitamin E in general in preventing and treating osteoarthritis (Chin and Ima-Nirwana, 2018). In a separate study, the researcher comprehensively assessed the existing research findings on the impact of tocotrienol on skeletal health in male osteoporosis animal models while also exploring its potential mechanisms as an anti-osteoporotic agent (Chin and Ima-Nirwana, 2019). Furthermore, there are also available reviews that explore the potential mechanisms of tocotrienol in mitigating glucocorticoid-induced osteoporosis, drawing from existing *in vivo* and *in vitro* findings (Ekeuku et al., 2022). In a more recent article published by Singh et al., they conducted a scoping review specifically focusing n the impact of tocotrienol on arthritis (Tejpal Singh et al., 2023). According to the solitary clinical trial uncovered in the existing body of literature, palm tocotrienol could potentially enhance joint function in individuals with osteoarthritis. The author’s conclusion highlights tocotrienols as a promising candidate for its potential role as an anti-arthritic agent (Tejpal Singh et al., 2023). Many recent review papers tend to have a narrower focus, concentrating on specific musculoskeletal conditions rather than addressing the broader spectrum of challenges related to musculoskeletal health. This review paper aims to comprehensively analyse the existing body of research on the preventive and therapeutic impacts of tocotrienols on age-related musculoskeletal challenges.

## 2 Methodology

A literature search was carried out using the keywords (“tocotrienol”) AND (“osteoarthritis” OR “rheumatoid arthritis” OR “psoriatic arthritis” OR “gout” OR “low back pain” OR “osteoporosis” OR “sarcopenia”) in the Pubmed and Scopus databases in September 2023. Only original research articles published in English within the past decade were considered for inclusion, with unanimous agreement among all the authors regarding the selection of articles in this review.

## 3 Musculoskeletal changes in ageing

This section elaborates on the natural ageing process and delves into the multifaceted changes that unfold within bones, muscles and joints. These changes encompass alterations in bone mineral density, muscular mass and strength, as well as joint structures, collectively contributing to a decline in musculoskeletal health and functionality. The preservation of bone strength and density throughout ageing is intricately governed by the perpetual process of balanced skeletal remodelling. However, this equilibrium is perturbed within the ageing bone microenvironment, disrupting the harmonious interplay of bone formation and resorption (Roberts et al., 2016).

Osteoblasts, responsible for bone formation, originate from mesenchymal stem cells (MSCs). These versatile MSCs can differentiate into various cell lineages, such as chondrocytes, myocytes, adipocytes, and fibroblasts, contingent upon the specific cues present in their microenvironment (Chin et al., 2022). As a result, changes in both the quantity and functionality of MSCs can impact various musculoskeletal tissues by influencing the population of precursor cells, particularly osteoblasts. Notably, MSCs obtained from aged individuals demonstrated diminished ability for *in vitro* expansion, displaying a flattened and extensively spread morphology in contrast to the vigorously proliferating, elongated cells derived from younger donors (Colter et al., 2001; Baxter et al., 2004). An elevated presence of cytoplasmic granulation was likewise identified, as reported by Bonab, et al. This finding implies a potential accrual of protein waste materials, a phenomenon commonly associated with compromised autophagy, which is another mechanism associated with ageing process (Bonab et al., 2006).

In joints, the potential for bone-to-bone contact is prevented by several key factors, including the presence of cushioning cartilage lining the joints (referred to as articular cartilage), the encasement of joints by synovial membranes, and the presence of lubricating synovial fluid within the joints. As individual age, the pliability and range of motion in joints diminish due to a decrease in the volume of synovial fluid within the joints and the thinning of cartilage. Additionally, ligaments tend to shorten and lose flexibility, which further contributes to the perception of joint stiffness (Better Health Channel, 2015).

A spectrum of modifications is evident in ageing muscles, encompassing natural processes like age-related sarcopenia and pathological conditions such as cancer-related anorexia and cachexia syndrome. Age-related sarcopenia, marked by a decrease in muscle mass, is typically defined as having a muscle mass measurement that falls below two standard deviations when compared to a control group of individuals of the same sex aged 18 to 40. However, due to the lack of a precise definition, the prevalence of age-related sarcopenia has varied significantly, ranging from 8% to 40% (Abellan van Kan, 2009). In 2010, the European Working Group on Sarcopenia in Older People introduced a classification encompassing three distinct stages of this progressive phenomenon (Mitchell et al., 2012). These stages consist of presarcopenia, which is primarily marked by a decrease in muscle mass; sarcopenia, which involved both muscle loss and a reduction in strength or physical performance; and severe sarcopenia, which becomes apparent when muscle loss is accompanied by significant declines in both power and physical performance.

This contrast is distinct from cachexia, a multifaceted metabolic syndrome invariably linked to an underlying illness or inflammatory state, precipitating the simultaneous loss of muscle and fat mass. Cachexia entails elevated muscle protein synthesis and breakdown, elevated basal metabolic rate, heightened energy expenditure, inflammation and insulin resistance. However, clinicians may encounter challenges in discerning whether the tissue loss stems from sarcopenia or cachexia (Rolland et al., 2011). In any case, the outcome entails a reduction in muscle mass, strength and overall functionality (Bozzetti and Mariani, 2009; Evans, 2010).

The capacity for movement has been established as a fundamental factor in shaping the health and quality of life of the elderly population (Groessl et al., 2007; Trombetti et al., 2016). Insufficient physical activities have been demonstrated to diminish cognitive capabilities, limit self-reliance, and elevate the likelihood of experiencing fractures, falls and mortality (Campbell et al., 2013; Khalaj et al., 2014). Moreover, older adults who experience a decline in mobility have been noted to exhibit elevated morbidity, mortality, disability, hospitalisations, healthcare utilisation, and associated expenses (Shumway-Cook et al., 2002; Newman et al., 2006; Hardy et al., 2011). The factors contributing to restricted mobility are complex and vary. On one hand, a patient’s accumulation of illnesses and concurrent conditions can impact their ability to move. For example, prevalent factors like arthritis and knee osteoarthritis often play a role (Khalaj et al., 2014). On the other hand, a patient’s mobility is not just the sum of separate disease processes; rather, the relationship between anatomical or biochemical irregularities, physical signs, and mobility function often exhibits a nonlinear pattern (Sakthivadivel et al., 2022). Past studies have indicated that older individuals within the elderly age group who show enhanced mobility often experience an improved quality of life. Conditions that hinder mobility, such as arthritis, are likely to have an impact on the overall quality of life (Trombetti et al., 2016; Shafrin et al., 2017).

The need for interventions for these age-related issues is crucial for several reasons. The decline of physical and cognitive functions due to ageing impacts an individual’s independence and quality of life. These issues strain healthcare systems, leading to higher costs and resource allocation. Addressing them can ease the burdens of the caregiver, benefit society’s productivity, and leverage medical advancements. Ultimately, these interventions enable dignified ageing, preserving independence and wellbeing.

## 4 Musculoskeletal diseases

Osteoarthritis (OA), which stands as the most common type of arthritis (Mora et al., 2018), is a degenerative joint disorder that arises from the gradual breakdown of the tissues that act as a cushion between the ends of bones within the joints (National Insitute on Aging, 2022). The most often afflicted joints in OA are the knee and hip joints. It has been estimated that knee pain affected 30.8% of individuals in Kuala Lumpur, Malaysia, who were 55 years and older, whereas self-reported symptoms of OA affected 25.4% of the population (Mat et al., 2019). In the past, it was believed that OA was caused by “wear and tear” of articular cartilage due to constant mechanical stress. Nevertheless, current insights indicate that OA is a dynamic response to injury, involving the remodelling of articular cartilage and subchondral bone, as well as inflammation of the synovial tissue, and damage to other joint components such as ligaments and menisci (Goldring, 2012).

Rheumatoid arthritis (RA) is a chronic joint-specific auto-inflammatory condition. Although RA can affect individuals of any age, its prevalence continues to rise into the seventh decade (Crowson et al., 2011). Based on the 2019 Global Burden of Disease Study, RA posed a significant global public health challenge with an estimated 18.5 million prevalent cases, consistently higher prevalence in females, and a projected trend of increasing age-standardized incidence rates (Shi et al., 2023). RA exhibits a disproportionate impact on women, with incidence and prevalence rates that are twice as high as those in men. The lifetime risk of developing RA is 3.6% for women and 1.7% for men (Crowson et al., 2011). As life expectance rises, the number of elderly individuals with RA grows, necessitating the development of novel ways to maximise treatment in this group.

Psoriatic arthritis (PsA) is an inflammatory disorder that is prevalent in about 0.8% of the general population (Alinaghi et al., 2019). This chronic, inflammatory disease affects the joints and entheses, where tendons and ligaments are connected to the bones. PsA is associated with similar health issues as psoriasis. It can manifest at any age, including youngsters. Typically, the disease appears between the ages of 30 and 50. Many people acquire PsA around 10 years after acquiring psoriasis, although some develop it before or without experiencing or detecting psoriasis (National Psoriasis Foundation, 2022). Although there is no cure, many treatments are available to halt the progression of the disease, reduce pain, protect joints, and preserve range of motion.

Gout is an inflammatory musculoskeletal condition with an incidence of between 1.6% and 6.8% (Day et al., 2019). This condition is defined by episodes of intense joint pain triggered by high levels of uric acid in the bloodstream and the accumulation of monosodium urate crystals in the joints. Recurring attacks can adversely impact the quality of life and lead to irreversible joint damage (Day et al., 2019). The frequency of gout increases with age, while age-related renal impairment increases with age.

Lower back pain (LBP) is the most prevalent health issue, resulting in pain and impairment in older persons (Bressler et al., 1999; Hoy et al., 2012). Individuals aged 65 years or older represent the second most common age group seeking medical attention for lower back pain (Cypress, 1983). Recent research indicates that lower back pain remains a prevalent issue among older adults who have reached retirement age (Fernández-de-las-Peñas et al., 2011; Fernández-de-Las-Peñas et al., 2013). Globally, the 1-year prevalence of lower back pain among seniors residing in communities varied from 13% to 50% (Patel et al., 2013; Leopoldino et al., 2016). Similarly, up to 80% of senior citizens living in long-term care facilities report having significant musculoskeletal pain (Tarzian and Hoffmann, 2005), and LBP accounts for one-third of these instances (D'Astolfo and Humphreys, 2006).

Osteoporosis, which means “porous bones” in Latin, is characterised by bone quality and density loss. The risk of fracture increases as the bone becomes more porous and brittle. Since the loss of one density and quality generally occurs silently and gradually, there are frequently no symptoms until the first fracture (National Institute of Arthritis and Musculoskeletal and Skin Diseases, 2019). Osteoporosis is one of the non-communicable illnesses that is rapidly spreading around the world. Fragile fractures most frequently found in the wrist, hip and spine are the results of osteoporosis. It is estimated that about 10 million people in the United States have osteoporosis (Better Health Channel, 2015). There is a significant healthcare cost for people, families and society because of increasing illness and death (Zha et al., 2015).

Sarcopenia is a kind of muscular failure brought on by adverse muscle changes accumulated over a lifetime and is typically observed in older people (Cruz-Jentoft et al., 2019). According to research, between 5% and 13% of older persons between 60 and 70 years old have sarcopenia. Furthermore, it was observed that individuals aged 80 years and older exhibited a sarcopenia prevalence ranging from 11% to 50%. (von Haehling et al., 2010). In Malaysia, nearly 40% of Malaysia’s older population experience cognitive pre-frailty and cognitive frailty. Additionally, 3 to 4 out of every 10 elderly individuals in Malaysia develop sarcopenia, which is characterized by the age-related loss of muscle mass. (Malek Rivan et al., 2019).

## 5 Sources, properties and bioavailability of tocotrienols

Tocotrienol is a lipid-soluble molecule belonging to the vitamin E family. It holds a significant sway over diverse biological processes. With their unsaturated phytyl chain isoprenoids, tocotrienol possesses a structural advantage. This distinctive architecture enables them to navigate through saturated fatty tissue layers efficiently, enhancing their bioavailability and potential impact (Chakraborty et al., 2014). Within its core structure, tocotrienol harbours three double bonds, strategically positioned at its hydrocarbon tail’s 3′, 7′ and 11′ sites. These double bonds enhance molecular fluidity, facilitating seamless integration into cellular membranes. This property closely resembles that of edible oils rich in polyunsaturated fatty acids, streamlining the absorption process within the body (Srikaeo, 2014).

**FIGURE 1:**
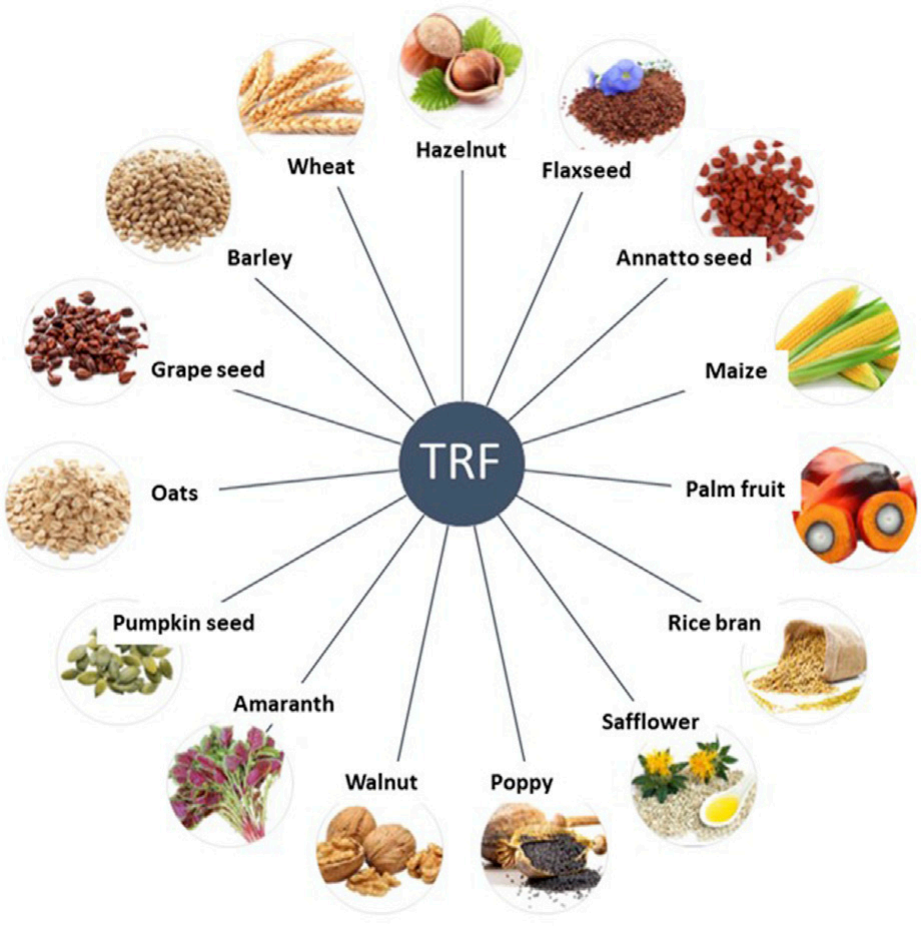
Natural sources of tocotrienols. Adapted from (Kannappan et al., 2012)

Tocotrienols are present in specific grains and plant-based sources like palm oil, rice bran oil, coconut oil, barley germ, wheat germ and annatto (Tan and Brzuskiewicz, 1989; Qureshi et al., 2000). Palm oil and rice bran oil stand out with notably tocotrienol levels, measuring 940 mg/kg and 465 mg/kg, respectively (Aggarwal et al., 2010). Tocotrienols are also sourced from grapefruit seed oil, oats, hazelnuts, maize, olive oil, Buckthorn berry, rye, flaxseed oil, poppy seed oil and sunflower oil (Figure 1) (Kannappan et al., 2011). Tocotrienols are found in different quantities within photosynthetic plants, and vegetable oils like sunflower, corn, safflower, and cottonseed serve as valuable reservoirs of these vitamin E isomers (Ahsan et al., 2015).

Comprehensive research indicates that tocotrienols demonstrate intriguing biological functions distinct from tocopherols. These encompass neuroprotective, radioprotective, anti-cancer, anti-inflammatory and lipid-lowering properties (Khanna et al., 2006; Nesaretnam, 2008; Nesaretnam et al., 2012; Selvaduray et al., 2012; Loganathan et al., 2013a; Loganathan et al., 2013b; Ray et al., 2013). Nonetheless, tocotrienols continue to receive less scientific attention when compared to more widely recognized tocopherol form of vitamin E. This disparity can partly be attributed to the scarcity of tocotrienols in dietary sources. Remarkably, the general public may not be aware that up to 70% of vitamin E in unprocessed palm oil is composed of tocotrienol. Annatto is recognized as the predominant source of δ-tocotrienol. l (Chooa et al., 2004; Ng et al., 2004; Schauss et al., 2013).

Similar to other vitamin E compounds, tocotrienols are absorbed in the small intestine in the presence of dietary fat. This process is facilitated by the enzyme esterase located in the stomach lining, which necessitates the presence of bile ducts. This absorbed form is then packaged into chylomicrons, subsequently entering the lymphatic system; *α*-tocotrienol is more efficiently absorbed than other tocotrienol forms. Tocotrienols primarily exert their antioxidant effects within the bloodstream by neutralizing harmful free radicals. Tissue uptake occurs through lipoprotein lipases that degrade lipoproteins into remnant particles, absorbed by the liver and peripheral tissues through a process involving receptor-mediated endocytosis. Tocotrienols are distributed across diverse tissue types, with the most significant concentrations found in adipose tissue and adrenal glands. Vitamin E, owing to its sluggish turnover rate, can be stored in tissues for extended periods. Following its antioxidant role, vitamin E undergoes oxidation and is converted to its hydroquinone form via a P450-dependent process before excretion via faeces. The hydroquinone form is combined with glucuronic acid and mixed with bile for removal. Despite tocotrienol’s promising potential, research has thus far explored only a fraction of vitamin E’s diverse capabilities. Nonetheless, biologists are progressively recognising the significance of this less prevalent yet distinctive vitamin E isomer (Hensley et al., 2004).

Regarding bioavailability, strong evidence indicates that tocotrienols can be found at notable levels in the bloodstream after both short-term and long-term supplementation. Nevertheless, there is a notable lack of sufficient data regarding the reference range of plasma tocotrienol concentrations required to produce significant physiological effects. While the pharmacokinetics of tocotrienols differ significantly from the extensively studied tocopherols, which have longer circulation times, biodistribution studies reveal a substantial accumulation of tocotrienols in vital organs. When it comes to therapeutic effectiveness, it is evident that the outcomes of clinical assessments are influenced by both the bioavailability of tocotrienols and the methodologies employed in the studies (Fu et al., 2014).

## 6 Effects of tocotrienol on musculoskeletal health

The predominant roles of tocotrienols in animals revolve around their antioxidant attributes. They thwart non-enzymatic oxidations of cellular constituents, such as unsaturated fatty acids, brought about by molecular oxygen and free radicals, including superoxide (O_2_^−^) and hydrogen peroxide (H_2_O_2_). The diverse biochemical functions of tocotrienols are intricately connected to their antioxidant prowess, contributing either directly or indirectly. Crucially, they play a pivotal role in maintaining the integrity and structure of cellular membranes (Atkinson et al., 2008). Tocotrienols encompass an extensive array of medicinal attributes and find application as antioxidants, analgesics, anti-inflammatories, antibacterials, antipyretics, antithrombotic, anticancer, cardioprotective, hepatoprotective, hypoglycaemics, and nephroprotective agents (Ahsan et al., 2014).

Free radicals and reactive oxygen species (ROS) encompass active molecules like superoxide anions, hydrogen peroxide, and hydroxide ions. These molecules emerge as natural byproducts during oxygen’s interaction in aerobic respiration and cellular metabolism within our body (Nimse and Pal, 2015). Nonetheless, when this equilibrium is disturbed, excessive reactive species can overpower the body’s natural antioxidant defences, resulting in oxidative stress. This condition adversely impacts lipids, proteins, and deoxyribonucleic acid (DNA) (Domazetovic et al., 2017). The human body has a built-in antioxidant defence system that efficiently scavenges free radicals, effectively reducing the levels of ROS. (Kawamura and Muraoka, 2018). Various factors, including ageing, smoking, alcohol consumption, intense physical activity, UV radiation exposure, and inadequate intake of antioxidants, can undermine the effectiveness of this defence system (Bhattacharyya et al., 2014; Liguori et al., 2018; Sharifi-Rad et al., 2020). Inhibition of antioxidant enzymes can trigger ROS buildup, which subsequently contributes to the emergence of bone disorders, decreased bone density (Sheweita and Khoshhal, 2007), and induced muscle damage and oxidative stress markers (Canals-Garzón et al., 2022). Hence, the introduction of an external antioxidant becomes essential due to its capacity to counterbalance oxidative stress efficiently (Srivastava, 2021) and becomes imperative as well as holds the potential for preventing diseases (Fiedor and Burda, 2014; Srivastava, 2021).

After an injury, satellite cells are activated and transform into myoblasts that undergo substantial proliferation initially. Around 3–7 days post-injury, myoblasts cease proliferation, transitioning to differentiation. They either fuse with damaged myofibers or amalgamate with each other to create myotubes, which are early-stage myofibers. Over the weeks, these nascent myofibers mature and develop into fully formed myofibers. In mouse models, this remarkably efficient process highlights the impressive regenerative capacity of skeletal muscles. They can substantially restore their structure and function within a few weeks after experiencing severe injury (Relaix and Zammit, 2012). While human skeletal muscles possess regenerative potential, they may not be as efficient as murine muscles. Research indicates that alterations in muscle morphology can persist for an extended period after an injury (Heiderscheit et al., 2010). In both animal and human scenarios, muscle injuries initiate an inflammatory response characterized by the coordinated influx of inflammatory cells to the site of injury (Smith et al., 2008). The commencement, development and eventual resolution of the inflammatory process have a vital role in shaping the functionality of satellite cells. This, in turn, has an impact on the muscle regeneration process (Tidball, 2005).

## 7 Tocotrienol in the prevention and treatment of musculoskeletal diseases

### 7.1 Osteoarthritis

Table 1 summarises studies investigating the impacts of tocotrienols on osteoporosis in both *in vitro* and *in vivo* studies. An *in vitro* study assessed and compared the chondroprotective effect of annatto tocotrienol and palm TRF using SW1353 chondrocytes treated with monosodium iodoacetate (MIA) demonstrated that both types of tocotrienols counteracted the decrease in chondrocyte viability caused by MIA exposure. Interestingly, cotreatment of annatto tocotrienol and MIA suppressed 8-isoprostane F2-α levels, an oxidative stress marker that enhanced the type II collagen/type I collagen ratio (Pang et al., 2021). Moreover, annatto tocotrienol co-exposure with MIA prompted upregulation of SOX9, type II collagen, and aggrecan levels, indicating potential self-repair and anabolic effects on chondrocytes exposed to MIA (Pang et al., 2021).

**TABLE 1:** Summary of studies of tocotrienols on osteoarthritis.

Study	Tocotrienol	Study model	Results
*In vitro*			
Pang et al. (2021)	Annatto tocotrienol and palm tocotrienol-rich fraction	SW1353 chondrocytes incubated with both tocotrienols alongside MIA in advance for 24 h	↓ 8-isoprostane F2-α
			↑ type II collagen/type I collagen ratio
			↑ SOX9, type II collagen and aggrecan levels
***In vivo*** (***animal***)			
Al-Saadi et al. (2021)	Palm tocotrienol	Male Sprague-Dawley (3 months-old, 250–300 g). 6 rats per group. Rats induced with osteoarthritis via intra-articular injection of MIA at the right knee. Rats were given TRF at 100 mg/kg/day for 4 weeks	↑ body weight
			Improved grip strength
			↓ serum COMP
Chin et al. (2019)	Annatto tocotrienol	Male Sprague Dawley rats (3-months-old). 4 groups were induced with osteoarthritis via single injection of MIA at week 0. 3 groups received annatto tocotrienol at 50, 100 and 150 mg/kg/day for 5 weeks	↓ histological scores and cartilage remodeling markers
			↓ osteocalcin and osteoclast surface of subchondral bone
***In vivo*** (***human***)			
Haflah et al. (2009)	Palm vitamin E	79 patients recruited at UKMMC received either 1.5 g oral glucosamine sulphate or 400 mg oral palm vitamin E for 6 months	Significant improvement in the WOMAC scale
			↓ VAS score
			↓ serum malondialdehyde
			↑ serum vitamin E

Abbreviations: Monosodium iodoacetate (MIA); sex-determining region Y box protein (SOX9); alkaline phosphatase (ALP); transforming growth factor beta (TGF-β); matrix metalloproteinase-2 (MMP-2); muscle segment homebox 2 (Msx2); bone morphogenetic protein 1 (BMP1); vascular endothelial growth factor beta (VEGF-β); dentin sialophosphoprotein (DSPP); cartilage oligomeric matrix protein (COMP); Runt-related transcription factor 2 (RUNX2); fibroblast growth factor receptor 3 (FGFR3); osteopetrosis-associated transmembrane protein 1 (Ostm1); microphthalmia-associated transcription factor (MITF); epidermal growth factor receptor (EGFR); Western Ontario and McMaster Universities (WOMAC); visual analogue scale (VAS). ↑ - increased or upregulated; ↓ - decreased or downregulated.

In a preclinical study, adult male Sprague-Dawley rats were treated with palm tocotrienol-rich fraction (TRF) at 100 mg/kg for 4 weeks (Al-Saadi et al., 2021). The results indicated a significant rebound in body weight for rats receiving treatment after an initial reduction. The group of rats receiving combined treatments of TRF and glucosamine showed notably improved grip strength compared to both the individual treatments. Serum cartilage oligomeric matrix protein (COMP) levels were lower in all treated groups compared to the control group (Al-Saadi et al., 2021). A separate study investigating the effects of 150 mg/kg/day oral annatto tocotrienol supplementation on MIA-induced Sprague Dawley rat models of osteoarthritis reported a significant reduction of osteocalcin levels and osteoclast surface in the subchondral bone (Chin et al., 2019), suggesting that annatto tocotrienol could potentially slow down osteoarthritis progression.

In an *in vivo* human study, 64 participants completed the trial with 6 months of treatment with palm vitamin E (Haflah et al., 2009). In this research, a noticeable distinction was observed in the serum levels of malondialdehyde (MDA) in the glucosamine group compared to the palm vitamin E group. MDA, generated as the final product of cellular peroxidation of polyunsaturated fatty acids, is a widely recognized marker indicating both oxidative stress and the body’s antioxidant capabilities (Cui et al., 2018). The significance of MDA as a biomarker for lipid peroxidation in a range of disease contexts and animal models is primarily attributed to its practicality and ease of detection (Merino de Paz et al., 2023). Additionally, serum vitamin E levels were notably higher in the palm vitamin E group compared to the glucosamine group. This study suggests that daily oral supplementation of 400 mg palm vitamin E potentially alleviates knee osteoarthritis symptoms (Haflah et al., 2009). Nevertheless, it is important to acknowledge that there is a scarcity of recent human studies examining the impact of tocotrienol on osteoarthritis. Additional research in this field is needed to offer more current and comprehensive perspectives on the potential therapeutic use of tocotrienol in the management of osteoarthritis.

### 7.2 Rheumatoid arthritis

Table 2 summarises studies investigating the impacts of tocotrienols on rheumatoid arthritis in both *in vitro* and *in vivo* studies. In an *in vitro* experiment, the research examined the impact of tocotrienol on IL-17-induced RANKL production in fibroblast-like synoviocytes (FLS) from rheumatoid arthritis (RA) patients and on osteoclast differentiation. The results demonstrated that tocotrienol reduced the production of RANKL induced by IL-17 in FLS and inhibited the formation of osteoclasts from monocytes exposed to IL-17, RANKL, IL-17-treated FLS, or Th17 cells. Additionally, it suppressed the differentiation of Th17 cells and the production of both IL-17 and soluble RANKL. (Kim et al., 2021).

**TABLE 2:** Summary of studies of tocotrienols on rheumatoid arthritis.

Study	Tocotrienol	Study model	Results
*In vitro*			
Kim et al. (2021)	Tocotrienol	Human PBMCs were cultured for 48 h with stimuli to induce Th17 differentiation. Tocotrienol was cultured with PBMCs for 3 h before subjecting them to the same Th17 differentiation method. RA FLS were stimulated with IL-17. The FLS were cultured with or without tocotrienol for 3 h before the addition of IL-17. After stimulation for 72 h, mRNA was extracted.	↓ IL-17-activated expression of RANKL
			↓ TNF-α level
			IL-6 and IL8 unchanged
***In vivo*** (***animal***)			
Zainal et al. (2019)	TRF	Female Dark Agouti rats induced with arthritis through single intradermal injection of collagen type II. Starting from day 28 after initial collagen injection, the rats were treated with TRF via oral gavage	↓ articular index scores, ankle circumferences, paw volumes and radiographic scores
			↓ plasma C-reactive protein levels and pro-inflammatory cytokines
			↓ severity of histopathological changes
Haleagrahara et al. (2014)	δ-tocotrienol	Female Dark Agouti rats (6–10 weeks old, 150–200 g). 6 rats in each group. Arthritis was induced by intradermal injection of collagen type II emulsified in complete Freund’s adjuvant and treated with 10 mg/kg tocotrienol from day 25–50	↓ paw oedema
			Significantly reversed the histopathological changes
Radhakrishnan et al. (2014)	γ-tocotrienol	Female Dark Agouti rats (10 weeks old, 120–140 g). Induced with arthritis using intradermal injection of collagen type II emulsified in complete Freund’s adjuvant. Subsequently treated with oral tocotrienol at a dose of 5 mg/kg, starting from day 21 through daily gavage until day 45	↓ body weight
			↓ CRP, TNF-α, SOD and the total GSH levels
			↓ histopathological changes
			Significant antioxidant and anti-inflammatory effect
Ahmed Ahmed (2020)	TRF	Albino Wistar rats administered TRF daily, starting from day 30 until day 45 after induction of TMJ arthritis	Mild inflammatory changes
			↑ BMD
***In vivo*** (***human***)			
-	-	-	-

Abbreviations: Interleukin-17 (IL-17); receptor activator of nuclear factor kappa beta ligand (RANKL); C-reactive protein (CRP); tumour necrosis factor-alpha (TNF-α), superoxide dismutase (SOD); glutathione (GSH); temporomandibular joint (TMJ); bone mineral density (BMD). ↑ - increased or upregulated; ↓ - decreased or downregulated.

In an animal study conducted by Zainal et al., female Dark Agouti rats were induced with arthritis by intradermally injecting collagen type II and subsequently treated with TRF via oral gavage, starting from day 28 after the initial induction of arthritis (Zainal et al., 2019). The study indicated that rats given TRF supplementation experienced noteworthy reductions in various measures, such as articular index scores, ankle circumferences, paw volumes, and radiographic scores, in contrast to rats that did not receive TRF. The untreated rats exhibited higher levels of plasma C-reactive protein and the production of pro-inflammatory cytokines compared to the TRF-fed rats. Furthermore, histopathological examination demonstrated a significant reduction in the severity of observed changes in the rats treated with TRF in comparison to the untreated rats. (Zainal et al., 2019). In a parallel study featuring female Dark Agouti rats with collagen-induced arthritis (CIA), the investigator noted that administering 10 mg/kg of tocotrienol from day 25 to day 50 resulted in a more substantial decrease in paw oedema compared to the use of glucosamine (Haleagrahara et al., 2014). The alterations in paw oedema observed in this study were in line with the histopathological findings, demonstrating a notable reversal of changes in the groups treated with tocotrienol. These findings suggest that tocotrienol treatment was markedly more effective in reducing paw oedema and reversing the histological changes associated with CIA in these rats when compared to glucosamine (Haleagrahara et al., 2014). In a closely related study, adult female Dark Agouti arthritis models received oral administration of 5 mg/kg body weight of γ-tocotrienol from day 21 to day 45. The results demonstrated that tocotrienol treatment significantly mitigated the arthritis-induced alterations in body weight, CRP, TNF-α, SOD, and total GSH levels. Additionally, histopathological changes induced by arthritis were substantially reduced with *γ*-tocotrienol treatment. (Radhakrishnan et al., 2014). In a recent study aimed at investigating the therapeutic potential of TRF in the context of temporomandibular joint (TMJ) arthritis, Albino Wistar rats received daily TRF treatment from day 30 to day 45 after induction of TMJ arthritis. The results indicated mild-inflammatory changes in TMJ histopathology in the TRF-treated group. Furthermore, the TRF-treated group showed an increase in bone mineral density following TRF administration. (Ahmed Ahmed, 2020).

### 7.3 Osteoporosis

Table 3 summarises studies investigating the impacts of tocotrienols on osteoporosis in both *in vitro* and *in vivo* studies. An *in vitro* study where preosteoblast MC3T3-E1 cells were treated with varying annatto tocotrienol concentrations for 24 days showed that annatto tocotrienol notably increased the expression of osteoblastic differentiation markers (OSX, COL1α1, ALP, OCN) and alkaline phosphatase (ALP) activity in a time-dependent manner (Wan Hasan et al., 2018). Moreover, annatto tocotrienol-treated groups exhibited enhanced collagen and mineralised nodule formation. This suggests that annatto tocotrienol promotes bone formation-related genes and proteins, indicating its positive influence on osteogenic activity (Wan Hasan et al., 2018). Another study reported that d-δ-tocotrienol induced mineralised nodule formation, alkaline phosphatase activity and differentiation marker gene expression in preosteoblast. It downregulated HMG CoA reductase, and most importantly, tocotrienol did not affect cell viability, suggesting its potential to suppress and maintain bone health (Shah and Yeganehjoo, 2019). Other than that, 68 genes related to osteogenesis and osteoclastogenesis were significantly upregulated such as alkaline phosphatase (ALP), transforming growth factor-beta 1 (TGF-β1) and runt-related transcription factor 2 (RUNX2). Conversely, specific genes like osteopetrosis-associated transmembrane protein 1 were downregulated (Ahn et al., 2011).

**TABLE 3:** Summary of studies of tocotrienols on osteoporosis.

Study	Tocotrienol	Study model	Results
*In vitro*			
Wan Hasan et al. (2018)	Annatto tocotrienol	Murine MC3T3-E1 preosteoblastic cells were cultured in various concentrations of Annatto for 24 days	↑ OSX, COL1α1, ALP and OCN
			↑ Type I collagen from day 3 to day 15
			↑ ALP activity from day 9 to day 21
Shah and Yeganehjoo (2019)	*d*-δ-tocotrienol	Murine MC3T3-E1 cells were exposed to tocotrienol (5–25 umol/L), tocopherol (25 umol/L), and lovastatin (0.1 umol/L) in the differentiation medium	↑ alkaline phosphatase activity
			↑ calcium deposition
			↑ BMP-2 expression
			↓ HMG-CoA reductase expression
Ahn et al. (2011)	Vitamin E	Human bone marrow stem cells cultured in osteogenic differentiation medium, and introduced to vitamin E	↑ cell proliferation
			↑ ALP, TGF-β, MMP2, Msx2, BMP1, biglycan, VEGF-β, DSPP, COMP, RUNX2, FGFR3, SMAD2
			↓ Ostm1, MITF, EGF
***In vivo*** (***animal***)			
Soelaiman et al. (2012)	Palm tocotrienol	Female Sprague-Dawley (4-month-old, about 180–200 g). 8 rats per group. Rats underwent ovariectomy and subjected to daily oral gavage of 60 mg/kg-1 of tocotrienol at 9 a.m., over period of 8 weeks	↑ bone formation by, ↑ dLS/BS, ↓ sLS/BS, ↑ MS/BS, ↑ MAR, ↑ BFR/BS
Muhammad et al. (2013)	Palm tocotrienol mixture	Female Wistar rats ovariectomised and given 60 mg/kg bw of tocotrienol via oral gavage 6 days/week for 4 weeks	↓ osteocalcin, IL1 and IL6
Deng et al. (2014)	γ-Tocotrienol	C57BL/6 female mice (8-week-old, 23–25 g). 8 mice per group. Mice underwent ovariectomy and were administered 100 mg/kg bw emulsified tocotrienol via subcutaneous injection once per month for 3 months	↑ BMD
			↑ BV/TV, Tb. Th, Tb. N and Tb.Sp
			↓ N.Oc
			↑ N.Ob
			↑ MAR, BFR
			↑ osteocalcin
			↓ CTX-1
Ibrahim et al. (2015)	Annatto tocotrienol	Female Sprague Dawley rats (200–250 g). Rats were ovariectomized and given 60 mg/kg annatto tocotrienol particles via injection for 4 weeks	↑ osteocalcin, BMP-2, VEGF-α and RUNX-2 (with lovastatin + tocotrienol treatment)
Mohd Ramli (2018)	Palm tocotrienol	Male Sprague Dawley rats (3-months-old, 300–320 g). 8 rats per group. Rats underwent adrenalectomy and supplemented with 60 mg/kg/day tocotrienol for 2 months	Maintained serum resorption marker level and preserved bone structure and strength
***In vivo*** (***human***)			
Shen et al. (2018)	Annatto seed (90% δ-tocotrienol +10% γ-tocotrienol)	89 postmenopausal osteopenic women (59.7 ± 6.8 years old, BMI 28.7 ± 5.7 kg/m^2^) supplemented with tocotrienol daily for 12 weeks	↓ NTX levels, sRANKL, RANKL/OPG ratio
			↓ urine 8-OHdG
			↑ BALP/NTX ratio

Abbreviations: Osterix (OSX); collagen-1-alpha (COL1α1); alkaline phosphatase (ALP); osteocalcin (OCN); double-labelled surface/bone surface (dLS/BS); single-labelled surface/bone surface (sLS/BS); mineralising surface/bone surface (MS/BS), mineral apposition rate (MAR); bone formation rate/bone surface (BFR/BS); interleukin-1 (IL-1); interleukin-6 (IL-6)); bone mineral density (BMD); bone volume normalised by tissue volume (BV/TV); trabecular number (Tb.N) and trabecular thickness (Tb.Th); trabecular separation (Tb.Sp); osteoclast number (N.Oc); osteoblast number (N.Ob); bone formation rate (BFR); Carboxyterminal cross-linking telopeptide type 1 collagen (CTX-1); bone morphogenetic protein (BMP-2); vascular endothelial growth factor (VEGF-α); Runt-related transcription factor (RUNX-2); N-terminal telopeptide (NTX); soluble receptor activator of nuclear factor-kappa B ligand (sRANKL); osteoprotegerin (OPG), 8-hydroxy-2′-deoxyguanosine (8-OHdG); bone-specific alkaline phosphatase (BALP). ↑ - increased or upregulated; ↓ - decreased or downregulated.

In an *in vivo* study conducted on ovariectomized female Sprague-Dawley rats, which were administered 60 mg/kg of palm tocotrienol for a duration of 8 weeks, the results indicated that tocotrienol significantly enhanced bone formation in estrogen-deficient rats. This was evident through an increase in the double-labelled surface/bone surface (dLS/BS), a decrease in the single-labelled surface/bone surface (sLS/BS), an increase in mineralizing surface/bone surface (MS/BS), an increase in mineral apposition rate (MAR), and an overall increase in bone formation rate/bone surface (BFR/BS). (Soelaiman et al., 2012). The study also concluded that tocotrienol was more effective than calcium in preventing bone loss due to estrogen deficiency. Another study examining the effects of tocotrienol supplementation in ovariectomised Wistar rats reported that tocotrienol supplementation decreased osteocalcin, IL-1 and IL-6 levels in ovariectomized rats, which suggests that tocotrienol countered the high bone turnover rat linked to estrogen deficiency, potentially serving as an anti-osteoporotic agent for postmenopausal women (Muhammad et al., 2013).

A study revealed noteworthy protection of bone health in mice when they were provided with 100 mg/kg of emulsified γ-tocotrienol through monthly subcutaneous injections for a duration of 3 months following ovariectomy (Deng et al., 2014). This was confirmed by improvements in bone structural parameters, changes in the expression of bone metabolic genes, and alterations in serum markers related to both bone resorption and formation (Deng et al., 2014). In a different study, ovariectomized female Sprague Dawley rats were either administered 60 mg/kg of tocotrienol particles or received a combined treatment of 750 ug/kg of lovastatin particles along with 60 mg/kg of tocotrienol via injections for a duration of 4 weeks. The findings showed that the group receiving that combined treatment exhibited notably higher gene expression of osteocalcin, BMP-2, VEGF-α, and RUNX-2 (Ibrahim et al., 2015). This shows that the concurrent administration of lovastatin and tocotrienol led to an upregulation of genes associated with fracture healing. A study by Mohd Ramli et al., involving male Sprague-Dawley rats that have undergone adrenalectomy and received oral palm tocotrienol at 60 mg/kg/day for 2 months, reported that long-term glucocorticoid treatment increased bone resorption and decreased bone strength (Mohd Ramli, 2018). Additionally, the expression of genes related to osteoblast and osteoclast activity showed increased bone turnover. The study also noted that the inclusion of palm tocotrienol in the diet helped sustained serum markers associated with bone resorption and maintained bone structure and strength. Furthermore, gene expression analysis revealed a decrease in bone resorption levels (Mohd Ramli, 2018).

Tocotrienol’s potential effects on osteoporosis have not yet undergone investigation in human trials. The existing body of research on the bone effects of tocotrienols in humans is limited, consisting of only two known studies. Specifically, these studies have focused on examining alpha-tocotrienol’s bone health protective properties in postmenopausal osteopenic women. In these studies, participants were randomised into distinct groups, receiving either placebo/olive oil, a low-dose tocotrienol (430 mg) or a high-dose of tocotrienol (860 mg) over 12 weeks Annatto tocotrienol. Encouragingly, significant positive outcomes were observed, including a notable reduction in urinary N-telopeptides (NTX) levels, soluble receptor activator of nuclear factor kappa-B ligand (sRANKL), and the soluble RANKL/osteoprotegerin (OPG) ratio. Additionally, improvements were seen in the bone alkaline phosphatase ALP/NTX ratio, and a decrease in urinary 8-hydroxy-2′deoxyguanosine levels was noted (Shen et al., 2018). In related research, the introduction of 600 mg of tocotrienol through supplementation over 12 weeks yielded notable results. This intervention triggered an increase in lysophospholipids while concurrently decreasing the levels of acylcarnitines and catabolites associated with tryptophan and steroids in postmenopausal women with osteopenia. These findings imply a potential dampening effect on inflammation and oxidative stress, offering promising implications for enhancing overall bone health (Shen et al., 2021).

### 7.4 Sarcopenia

Numerous studies have also explored the potential benefits of tocotrienols in addressing sarcopenia, a critical age-related decline in skeletal mass and function. Table 4 summarises the *in vitro* and *in vivo* studies of tocotrienols on sarcopenia. In an *in vitro* study conducted by Khor et al., primary human myoblasts were cultured at both young and senescent stages, and then exposed to TRF or α-tocopherol for 24 h. Both treatments proved beneficial for senescent myoblasts, as they restored a morphology similar to that of young cells, improved cell viability, and reduced the expression of SA-β-gal. Additionally, the administration of TRF distinctly increased BrdU incorporation, particularly in senescent myoblasts, and it facilitated myogenic differentiation by influencing MRFs (Myogenic Regulatory Factors) at both mRNA and protein levels (Khor et al., 2016). The findings highlight the superior efficacy of TRF compared to *α*-tocopherol in alleviating abnormalities associated with replicative senescence and promoting myoblast differentiation by influencing MRFs. This suggests the potential use of vitamin E in addressing replicative senescence in myoblasts.

**TABLE 4:** Summary of studies of tocotrienols on sarcopenia.

Study	Tocotrienol	Study model	Results
*In vitro*			
Khor et al. (2016)	TRF	Primary human myoblast cultured into young and senescent phase, treated with 50 μg/mL TRF for 24 h	Restore morphology of young cells
			Enhance cell viability
			↓ SA-β-gal expression
			↑ BrdU, MRFs
Lim et al. (2013)	TRF	Human myoblasts induced by SIPs and treated with different concentrations of TRF (0 μg/mL–250 μg/mL) for 24 h	↓ SA-β-gal activity
			↑ cell proliferation
Lim et al. (2019)	TRF	CHQ5B were treated with 50 μg/mL for 24 h after SIPS induction	↑ *EREG, SHC1, SHC3* (regulate ErbB)
			↓ *MSTN, SMAD3* (regulate FoxO)
			↔ p53 signalling, MRF, cell cycle, Wnt signalling pathways
***In vivo*** (***animal***)			
Saud Gany et al. (2022)	TRF	Male Sprague-Dawley rats (3 months old, 9 months old, 21 months old). Received 60 mg/kg/day TRF via oral gavage for 3 months	↑ lipid metabolism
			↑ energy and amino acid metabolism
			↑ amino acid synthesis and muscle regeneration
			↑ energy metabolism
Lee et al. (2009)	TRF	Male Wistar rats (6-weeks-old) on an isocaloric diet were orally administered either 25 mg/kg TRF or 50 mg/kg TRF, or 25 mg/kg D-α-tocopherol for 28 days. Followed by a forced swimming endurance test	↑ swimming time
			↑ SOD, CAT, GPx
			↓ blood lactate, TBARS, muscle protein carbonyl
***In vivo*** (***human***)			
-	-	-	-

Abbreviations: Tocotrienol-rich fraction (TRF); senescence-associated *ß*-galactosidase (SA-β-gal); bromodeoxyuridine (BrdU); myogenic regulatory factors (MRFs); stress-induced premature senescence (SIPS); human skeletal muscle myoblasts (CHQ5B); erythroblastic leukemia viral oncogene homologue (ErbB); Forkhead box transcription factors (FoxO); myogenic regulatory factor (MRF); Wingless and Int-1 (Wnt); thiobarbituric acid-reactive substances (TBARS); superoxide dismutase (SOD); catalase (CAT); glutathione peroxidase (GPx). ↑ - increased or upregulated; ↓ - decreased or downregulated.

Another study investigated TRF’s impact on myoblasts’ regenerative potential under stress-induced premature senescence (SIPS). TRF (50 μg/mL) induced the highest cell proliferation in this study (Lim et al., 2013). Additionally, posttreatment with TRF decreased SA-β-gal activity and increased cell proliferation, suggesting a potential reversal of ageing effects (Lim et al., 2013). In a follow-up study that was built upon previous research, CHQ5B cells were exposed to 50 ug/mL of TRF for 24 h after being induced with SIPS. The results showed that TRF treatment had significant regulatory impact on specific gene pathways in SIPS myoblasts compared to the SIPS control group. Notably, TRF treatment influenced the p53 (RRM2B, SESN1), ErbB (EREG, SHC1, SHC3), and FoxO (MSTN, SMAD3) signalling pathways. These findings suggest that TRF has the potential to affect the proliferation capacity of SIPS myoblasts by modulation the ErbB and FoxO pathways, while also preserving satellite cell renewal through the p53 signalling pathway, along with other relevant pathways such as MRF, cell cycle, and Wnt signalling. (Lim et al., 2019).

In a preclinical studies involving animal models, specifically in a study conducted by Gany et al., using male Sprague Dawley rats of different ages (3, 9 and 21 months), the rats were supplemented with 60 mg/kg body weight/day of TRF through oral gavage for a period of 3 months. The findings indicated that TRF treatment led to a significant upregulation of age-related lipid metabolism while downregulating energy and amino acid metabolism. In contrast, the groups receiving TRF supplementation exhibited an upregulation of metabolites related to energy and amino acid metabolism, including N6-methyl adenosine, spermine, phenylalanine, tryptophan, aspartic acid, histidine, and N-acetyl neuraminic acid. Furthermore, TRF supplementation improved energy metabolism, as evidenced by increased levels of nicotinamide adenine dinucleotide and glycerol 3-phosphate (Saud Gany et al., 2022). Overall, the study suggests that ageing skeletal muscle mass and function changes are linked to carbohydrate, lipid and amino acid metabolism. TRF supplementation enhanced energy and amino acid synthesis, potentially promoting skeletal muscle regeneration and renewal in ageing rats (Saud Gany et al., 2022).

**FIGURE 2:**
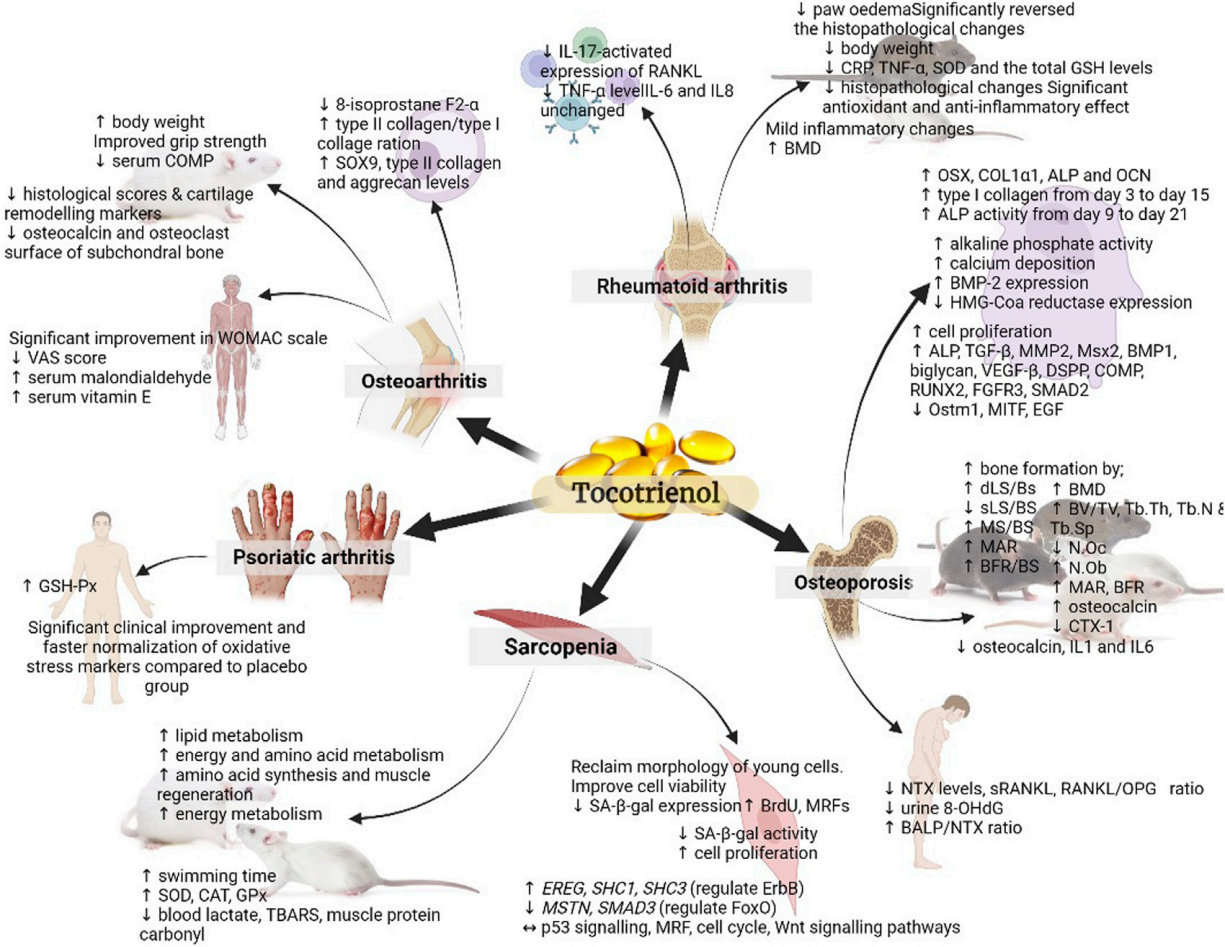
Summary of the role of tocotrienol in the prevention and treatment of musculoskeletal diseases. The figure presents a comprehensive visual representation of the research landscape for various musculoskeletal diseases. It highlights studies that have been conducted, such as those examining sarcopenia at the cellular and pre-clinical animal model levels, while also indicating areas where further investigation in human subjects is warranted. (↑: upregulation; ↓: downregulation; ↔: maintain).

In a separate study, rats were administered different doses of TRF (25 and 50 mg/kg) or D-α-tocopherol (25 mg/kg) orally for 28 days and were forced to swim. The TRF-treated rats exhibited significantly longer swimming times with TRF treatment. Also, they showed higher levels of liver and muscle glycogen, superoxide dismutase (SOD), catalase (CAT), and glutathione peroxidase (GPx), as well as lower levels of blood lactate and thiobarbituric acid-reactive substances (TBARS), and protein carbonyl (Lee et al., 2009). The study’s findings demonstrated that TRF supplementation can enhance endurance and reduce oxidative stress in rats undergoing forced swimming exercises. Although the study did not directly focus on sarcopenia, its results suggest that TRF may have a positive impact on muscle performance and oxidative stress, which are relevant factors in the context of sarcopenia, a condition characterised by muscle loss and decreased physical function often associated with ageing (Santilli et al., 2014). These outcomes suggest that TRF might have potential benefits for addressing aspects related to muscle health and performance, which could be relevant in sarcopenia management. Figure 2 provides a concise overview of how tocotrienols can be employed in both the prevention and treatment of musculoskeletal diseases.

### 7.5 Limitations of presently available treatments

In the realm of musculoskeletal disease management, while significant advancements have been achieved, various limitation persist. The predominant approach in many treatments often focuses on mitigating symptoms rather than addressing the root causes of these conditions, offering transient relief without necessarily yielding sustained amelioration. Recent comprehensive review of research has reported a myriad of medications and non-pharmacological treatments to relieve musculoskeletal pain conditions. Previous research has shown that there is substantial supporting evidence indicating that both exercise and psychosocial interventions are effective in alleviating pain and enhancing functionality in those conditions (Babatunde et al., 2017). According to guidelines from previous studies, non-pharmacological therapies should be the initial choice for addressing chronic low back pain and pain associated with osteoarthritis (Qaseem et al., 2017; Bannuru et al., 2019).

Non-steroidal anti-inflammatory drugs (NSAIDs), notably, have traditionally assumed a secondary or supplementary role in the pharmacological management of chronic musculoskeletal conditions. Prior to recent developments, the consensus among medical authorities advocated for opioid analgesics as a recourse for individuals experiencing chronic musculoskeletal pain when alternative interventions are ineffective. This counsel, disseminated extensively, led to the widespread and protractive utilization of opioids among a substantial portion of the chronic musculoskeletal patient population. Nevertheless, the landscape has shifted in light of evolving evidence, prompting a revision in recommendations. Opioids are presently excluded from the list of recommended treatments for chronic musculoskeletal pain disorders due to their lack of demonstrable superiority over alternative analgesic options (Busse et al., 2018; Krebs et al., 2018). Furthermore, the use of opioids carries a considerably heightened risk of severe adverse outcomes, encompassing addiction, physical harm, and even fatality (Dowell et al., 2016; Bannuru et al., 2019). The efficacy of certain treatments may vary across individuals and conditions, leaving some patients with insufficient relief. In cases where NSAID usage is contraindicated for patients, the primary therapeutic avenues often entail the utilization of intra-articular injections, encompassing corticosteroids or hyaluronic acid, as viable alternatives. These interventions exhibit the capacity to offer short-term relief from osteoarthritis-associated pain (Cheng et al., 2012; Bannuru et al., 2019). However, pharmacological interventions typically entail significant adverse effects, notably encompassing gastrointestinal irritation, haemorrhage, and diminished renal blood perfusion (Oray et al., 2016; Katz et al., 2021). Additionally, the progression of degenerative musculoskeletal diseases remains a challenge, particularly in cases of osteoarthritis, where joint deterioration continues despite therapeutic efforts (Walter and Frontera, 2018).

Surgical interventions, through at times indispensable, carry inherent risks and extended recovery periods. For instance, the orthopaedic implantable hardware, utilized in diverse scenarios ranging from joint replacement procedures to the management of traumatic injuries, has played a pivotal role in enhancing the quality of life for countless individuals (Kaufman et al., 2016). Among the prevalent challenges associated with procedures utilizing orthopaedic hardware, infection stands out as a primary concern. Joint infections, akin to foreign bodies, bestow an elevated vulnerability upon all patients to potential surgical site infections. These infections, when they occur, can yield catastrophic consequences, encompassing the loss of the affected extremities and, in certain dire instances, even the loss of life (Bozic et al., 2010; Bohm et al., 2012; Hernández-Vaquero et al., 2013).

Moreover, the prohibitive costs of certain treatments, like biologic drugs and advanced surgical procedures, can perpetuate healthcare disparities. Previous studies have estimated that prosthetic infections occur in a range from less than 1% of total hip and shoulder procedures to less than 2% of total knee procedures. It is important to note that these figures may not fully represent the true extent of the issue due to potential underreporting. However, infections have been identified as the leading cause of revision surgeries in a significant portion of cases, accounting for 14.8% of total hip arthroplasties and 25.2% of total knee arthroplasties. The overall cost of care associated with revision procedures represents a substantial financial burden, totalling approximately $50,000 (Bozic et al., 2010). While regenerative therapies such as stem cell treatments and platelet-rich plasma (PRP) injections, hold promise, their efficacy and long-term outcomes necessitate further investigation, limiting their applicability (Ramaswamy Reddy et al., 2018).

Many existing treatments primarily focus on symptom management rather than disease modification and tissue regeneration (National Academies of Sciences, Engineering, and Medicine et al., 2020; El-Tallawy et al., 2021). Musculoskeletal diseases often coincide with comorbidities (Slater et al., 2011), demanding a multidisciplinary approach. Patient compliance with prescribed regimens, encompassing exercise, medications, and lifestyle modifications, presents its own set of challenges, affecting treatment efficacy (Collado-Mateo et al., 2021). Recognizing and addressing these limitations is imperative in the management of musculoskeletal diseases, necessitating close collaboration with healthcare providers to determine the most suitable and efficacious treatment strategies for individual cases. Furthermore, ongoing research and evolving advancements in the field hold promise for improving treatment options and enhancing patient outcomes in the future.

## 8 Challenges and future directions

The current body of research on tocotrienols and their effects on musculoskeletal health reveals several notable research gaps and limitations. While some studies have explored the potential therapeutic role of tocotrienols in conditions such as osteoporosis, psoriatic arthritis, and sarcopenia, comprehensive human trials that specifically examine tocotrienol’s impact on these musculoskeletal disorders remain scarce. Most investigations have predominantly focused on α-tocopherols as representative of vitamin E, potentially overlooking the broader benefits and distinctive effects that different tocotrienol isomers might offer. Additionally, the existing studies on psoriatic arthritis utilised tocopherols, leaving an evident gap in our understanding of how tocotrienols could potentially influence this condition. Furthermore, the research landscape lacks an in-depth exploration of tocotrienols’ effects on other musculoskeletal diseases beyond the conditions mentioned.

To address the current gaps in understanding and exploring the mechanisms and benefits of tocotrienols in ageing-related musculoskeletal disorders, it is crucial to propose research directions that can shed light on their possible therapeutic roles. Well-designed, randomized controlled trials involving a diverse cohort of participants to investigate the effects of tocotrienol supplementation on ageing-related musculoskeletal disorders should be conducted. Clinically relevant outcomes, such as bone mineral density, joint function, muscle strength and overall quality of life, should be assessed in the trials.

It is also necessary to conduct head-to-head comparative studies between tocotrienol and standard treatments for musculoskeletal disorders. The efficacy of tocotrienol supplementation with existing interventions such as vitamin D and calcium supplementation, bisphosphonates, and other relevant therapies should be conducted. Other than that, longitudinal studies examining the long-term effects of tocotrienol supplementation on musculoskeletal health in the ageing population should be conducted. Changes in bone density, joint function, muscle mass, and functional abilities should be monitored over an extended period.

Another way to narrow the research gap is to employ molecular and cellular studies to elucidate the underlying mechanisms through which tocotrienols affect ageing-related musculoskeletal disorders. As of present, the pathways of interest relevant to tocotrienol’s action include inflammation, oxidative stress, cellular senescence, and signalling pathways relevant to bone health and muscle function. Also, the role of tocotrienols in promoting muscle regeneration and repair in the context of sarcopenia should be explored. The researchers should investigate tocotrienol’s potential to enhance satellite cell activation, myogenic differentiation, and overall muscle tissue repair. Besides that, the possibility of tocotrienols to support joint health and alleviate symptoms of osteoarthritis by studying their effects on cartilage integrity, inflammation and pain management in aged individuals should be studied.

Additionally, it is critical to utilise animal models that closely mimic ageing-related musculoskeletal disorders to gain insights into the preventive and therapeutic effects of tocotrienol supplementation. This can provide a holistic understanding of the impact of tocotrienols on musculoskeletal health in ageing populations. Another approach to address the research gap is to examine the influence of genetic factors on individual responses to tocotrienol supplementation for ageing-related musculoskeletal disorders by investigating genetic markers that might predict better outcomes. Lastly, nutrigenomic approaches should be used to uncover gene expression profiles and regulatory pathways influenced by tocotrienol supplementation in ageing-related musculoskeletal disorders.

By pursuing these research directions, we can further enhance our understanding of the mechanisms and potential benefits of tocotrienols in addressing ageing-related musculoskeletal disorders, contributing to developing effective preventive and therapeutic strategies.

## 9 Conclusion

The research investigating the effects of tocotrienols on musculoskeletal health has yielded key findings that underscore their potential therapeutic significance. Several studies have focused on the protective effects of tocotrienols on bone health, highlighting their ability to enhance bone mineral density, improve bone microarchitecture, and mitigate bone loss in various experimental models. Notably, tocotrienols have been shown to modulate bone turnover markers, promote osteoblast differentiation, and inhibit osteoclast activity, collectively suggesting their osteoprotective properties. Additionally, tocotrienols exhibit anti-inflammatory and antioxidant effects, essential in countering the oxidative stress and inflammation associated with musculoskeletal disorders. These attributes contribute to their potential to alleviate joint pain and inflammation in conditions such as osteoarthritis. Tocotrienols also exhibit promising effects on muscle health, showing the capacity to enhance muscle regeneration, counteract muscle atrophy, and improve muscle strength in both animal models and limited human studies.

In light of the promising findings and existing knowledge gaps, it is imperative to catalyse further research efforts to unlock the full potential of tocotrienols in addressing age-related musculoskeletal disorders. The insights from ongoing and future studies hold the key to shaping targeted and effective interventions that can alleviate the burden of conditions such as osteoporosis, sarcopenia and osteoarthritis. Rigorous and well-designed human clinical trials are essential to translate preclinical and mechanistic findings into clinically meaningful outcomes, guiding evidence-based practices for healthcare professionals and informing treatment strategies. By investing in comprehensive research, we can unravel the intricate mechanisms through which tocotrienols impact bone health, muscle regeneration and joint functions, thus paving the way for innovative therapeutic approaches. Collaborative efforts among researchers, clinicians and industry partners are paramount to derive this research agenda forward, bringing the current knowledge gaps and propelling tocotrienols from the laboratory to the clinical setting. As we unravel the potential benefits of tocotrienols, we have the opportunity to reshape the landscape of musculoskeletal health and offer new avenues of hope for individuals facing the challenges of age-related musculoskeletal disorders.
